# *Artemisia annua* Residue Regulates Immunity, Antioxidant Ability, Intestinal Barrier Function, and Microbial Structure in Weaned Piglets

**DOI:** 10.3390/ani14243569

**Published:** 2024-12-10

**Authors:** Jinjie Hu, Miaomiao Bai, Yueyao Xing, Junhong Liu, Kang Xu, Xia Xiong, Hongnan Liu, Yulong Yin

**Affiliations:** 1 College of Animal Science and Technology, Hunan Agricultural University, Changsha 410128, China; kinghu1987@126.com (J.H.); 18638009853@163.com (Y.X.); yinyulong@isa.ac.cn (Y.Y.); 2 Laboratory of Animal Nutritional Physiology and Metabolic Process, Key Laboratory of Agroecological Processes in Subtropical Region, Institute of Subtropical Agriculture, Chinese Academy of Sciences, Changsha 410125, China; xukang2020@163.com (K.X.); xx@isa.ac.cn (X.X.); 3 College of Animal Science and Technology, Northeast Agricultural University, Harbin 150030, China; 18832409659@163.com; 4 Hunan Provincial Key Laboratory of the Traditional Chinese Medicine Agricultural Biogenomics, Changsha Medical University, Changsha 410219, China

**Keywords:** *Artemisia annua* residue, immunity, intestinal barrier function, intestinal microbiota, weaned piglets

## Abstract

*Artemisia annua* residue (AR), as the byproduct of industrial extraction of artemisinin, contains rich nutrients and active ingredients. This study was conducted to determine the effects of AR as an unconventional feed material on growth performance, immunity, and intestinal health in weaned piglets. Thirty-two piglets weaned at 21 days (7.53 ± 0.31 kg average BW) were fed with a corn–soybean basal diet (BD) and a basal diet with 1% (LAR), 2% (MAR), and 4% (HAR) AR diets for 28 days. AR diets increased the serum IgA and complement component 3 levels, superoxide dismutase activity, and villus height in the duodenum (*p* < 0.05). The MAR group increased the ADG, serum total protein, and mRNA expression levels of Claudin-1 in the duodenum and zonula occludens-1 (ZO-1) and the mucin 2 (MUC2) in the colon, as well as colonic *Romboutsia* and *Anaerostipes* abundances, and decreased the *Proteobacteria* abundance (*p* < 0.05). To sum up, dietary AR supplementation may enhance growth performance by improving serum immunoglobulin and antioxidant enzyme activity, intestinal morphology, tight junction protein expression, and gut microbiota of weaned piglets. Regression analysis showed that the optimal AR supplemental level for growth performance, immunity, antioxidant ability, and intestinal health of weaned piglets was 2.08% to 4.24%.

## Simple Summary

*Artemisia annua* residue (AR), as the byproduct of the industrial extraction of artemisinin, contains rich nutrients and active ingredients, which have the potential to replace dietary corn or soybean meal and improve the production performance and intestinal health of livestock. This study found that dietary AR supplementation could increase serum antioxidant capacity, immunity, and intestinal barrier function to improve the growth performance of weaned piglets. Additionally, AR at an appropriate amount of 2.08~4.24% also improved the intestinal microbial structure, metabolic phenotypes, and short-chain fatty acid (SCFA) production, which benefits piglets by improving intestinal status and alleviating weaning stress. This contribution is theoretically and practically relevant because these findings can be applied in the livestock industry to alleviate weaning stress and improve intestinal health. Moreover, developing AR as a new feed material can broaden the comprehensive exploitation and utilization of *Artemisia annua* resources, improve the economic benefits, and relieve the shortage of feed resources and environmental pollution.

## 1. Introduction

In recent years, with the increasing demand for animal products, there has been a growing need for both adequate and inexpensive livestock feed in many developing countries. Combined with local feed resources and cost-effectiveness, the development of unconventional feed materials to partially replace grain concentrates as livestock feed contributes to the sustainable development of animal husbandry [1]. Moreover, unconventional feed materials contain various bioactive ingredients, which have several beneficial functions, such as antioxidant, and antibacterial activities, with a positive impact on animal production performance [2]. Therefore, improving the development and utilization level of unconventional feed resources to increase the supply of raw feed materials is our main research direction in the future.

*Artemisia annua* (*Artemisia annua* L.), a common traditional Chinese medicine, is planted in many countries. Research has demonstrated that *Artemisia annua* could be utilized as a new potential protein source for geese [3] and a potential supplement for improving growth performance and immune capacity for weaned piglets [4]. Moreover, *Artemisia annua* has the functions of promoting growth, enhancing immunity, and suppressing the occurrence of diseases in livestock production [5]. *Artemisia annua* residue (AR) is the byproduct of the industrial extraction of artemisinin, and the biomass yield of AR in China is approximately 1.6 × 10^4^ t per year. AR is rich in crude protein, crude fiber, amino acids, and other nutrients, and it has the characteristics of a wide activity range, high safety, and significant pharmacological function [6]. Moreover, AR contains a variety of active ingredients with antioxidant, anti-inflammatory, and antibacterial functions, mainly including flavonoids, volatile oils, and polysaccharides [7]. The methods of AR disposal are mostly burning or discarding it directly, and it is rarely used for agricultural fertilizer and livestock and poultry feeding, which not only causes environmental pollution but also causes the waste of resources in *Artemisia annua*. Developing AR as a new feed material can broaden the comprehensive exploitation and utilization of *Artemisia annua* resources, improve economic benefits, and relieve the shortage of feed resources and environmental pollution. Therefore, we speculated that AR could be used in livestock and poultry feed to enhance animal growth and health. However, the application of AR as an unconventional feed material in livestock and poultry production has rarely been reported. This study evaluated the potential of AR as an unconventional feed material for weaned piglets by examining the growth performance, immunity, antioxidant ability, and intestinal barrier function.

## 2. Materials and Methods

### 2.1. Animal Ethics Statement

All experimental programs and care standards were approved by the Chinese Academy of Science Institutional Animal Care Committee and Use Committee at the Institute of Subtropical Agriculture (Changsha, CAS20220050). Piglets in both experiments were housed in individual pens equipped with a controlled environment. Dry feed and clean water were available to all piglets.

### 2.2. Analysis of the Nutritional Value and Main Active Components of Artemisia annua Residue

AR was the byproduct of artemisinin extraction from *Artemisia annua* L. Dried and crushed AR was used for piglet feed. The nutritional value assessment of AR was performed. Moisture (method GB/T 6435-2014) [8], dry matter (method GB/T 6435-2014) [8], ash (method GB/T 6438-2007) [9], fat (method GB/T 6433-2006) [10], crude protein (method GB/T 6432-2018) [11], acid detergent lignin (method GB/T 20805-2006) [12], crude fiber (method GB/T 6434-2022) [13], neutral detergent fiber (method GB/T 20806-2022) [14], and acid detergent fiber (method NY/T 1459-2022) [15] were determined and are shown in Table 1.

Table 1 presents the primary active components of AR identified through High-Performance Liquid Chromatography (HPLC). (1) A mixture of 40 g of dried AR powder and 800 mL of 70% ethanol was extracted for 4 h in a water bath at 70 °C, cooled to room temperature naturally, and centrifuged for 10 min at 10,000× *g*, and the AR alcohol was separated. (2) A total of 100 g of dried AR powder was immersed in 1000 mL of distilled water for 12 h, then boiled for 1 h and filtered to obtain the first filtrate. In the same way, 1000 mL and 600 mL of distilled water were added for the second and third times, respectively; after pumping and filtering, the filtrate was combined with 3 parts and then concentrated by rotary evaporation and heating. Finally, the liquid was thickened and freeze-dried at −80 °C to separate the AR water extract. Freeze-dried AR alcohol and water extract was dissolved again in methanol solution by ultrasound, and the filtrate was combined twice and centrifuged at 5000× *g* for 10 min. The supernatant was filtered by a 0.22 µm filter membrane and then detected by ExionLC™ AC liquid chromatograph (SCIEX, Boston, MA, USA). Chromatographic conditions: a Kinnetex C18 (2.1 mm × 100 nm, 2.6 µm, Agilent, Santa Clara, CA, USA) column was used with 0.1% formic acid–acetonitrile as the mobile phase at the flow rate of 0.3 mL/min and a column temperature 40 °C, and the sample size was 2 µL.

### 2.3. Animal Experimental Details

Thirty-two castrated male weaned piglets (28 days old, Duroc × Large White × Landrace) weighing 7.50 ± 0.24 kg were divided into four groups. Each group was replicated eight times with one piglet per replicate. All piglets were healthy and rigorously subjected to the farm’s immunization program. Dietary treatments included the control group (BD); a basal diet without antibiotics; and a basal diet supplemented with 1.0% AR (LAR group), 2.0% AR (MAR group), and 4.0% AR (HAR group). The basal diet meets the NRC (2012) requirements for piglets weighing 7 to 11 kg, as shown in Table 2. The experimental period lasted 28 days. AR was directly added to the basal diet according to the proportion of each group and stirred evenly. The choice of AR dosage was based on previous studies on *Artemisia annua* L. and other plant materials [3,16]. During the experiment, all piglets were free to eat and drink.

### 2.4. Growth Performance

Piglets’ initial and final body weights, as well as daily intake feed, were accurately measured throughout the experiment. The average daily weight gain (ADG), average daily feed intake (ADFI), and feed/gain ratio for piglets were calculated using their weights along with feed disappearance.

### 2.5. Sample Collection

After fasting for 12 h, all piglets were weighed, anesthetized by intramuscular injection of 6 mg/kg BW ketamine, and slaughtered, and jugular vein blood samples were collected. The whole operation was carried out at the Institute of Subtropical Agriculture Ecology, Chinese Academy of Sciences. After standing at room temperature for 1 h, the collected whole blood samples were centrifuged at 3500× *g* at 4 °C for 10 min, and the supernatant was separated to obtain serum samples for the detection of serum biochemical, antioxidant, and immune indexes. And approximately 2 cm long segments were collected from the duodenum, jejunum, and ileum for histological analysis. After rinsing with pre-cooled saline, each intestinal segment (duodenum, jejunum, ileum, and colon) was collected and frozen with liquid nitrogen for molecular detection. A sterile enzyme-free centrifuge tube was used to collect 2 g of mid-colonic contents for further analysis at −80 °C.

### 2.6. Serum Biochemical Indices

The concentrations of total protein (TP, 03183734190), albumin (ALB, 03183688122), triglyceride (TG, 20767107322), glucose (GLU, 04404483190), cholesterol (CHOL, 03039773190), high-density lipoprotein (HDL-C, 04399803190), low-density lipoprotein (LDL-C, 03038866322), urea nitrogen (UN, 04460715190), and complement components 3 (C3, 03001938322) and 4 (C4, 03001962322), as well as the activities of aspartate aminotransferase (AST, 20764949322), alkaline phosphatase (ALP, 03333701190), and alanine aminotransferase (ALT, 20764957322) in serum, were analyzed using automatic biochemical analyzer (Beckman CX4; Roche Diagnostics GmbH, Mannheim, Germany) and Roche commercial kits (Roche, Basel, Switzerland).

### 2.7. Serum Immunoglobulin and Cytokine Concentrations

The serum immunoglobulin and cytokine levels were detected using commercial enzyme-linked immunosorbent assay kits (Jiangsu Meimian Industrial Co., Ltd., Yancheng, China). The concentrations of serum immunoglobulin (Ig) A (IgA), IgG, IgM, interleukin (IL)-1β, IL-6, and tumor necrosis factor-α (TNF-α) were measured following the manufacturer’s instructions and technical details: IgA (M1244L96)—the standard curve of 1 to 36 µg/mL and inter- and intra-assay CV of 12% and 10%; IgG (M1245L96)—the standard curve of 12 to 400 µg/mL and inter- and intra-assay CV of 12% and 10%; IgM (M1246L96)—the standard curve of 1.2 to 40 µg/mL and inter- and intra-assay CV of 12% and 10%; IL-1β (ml002302)—the standard curve of 1 to 45 ng/L and inter- and intra-assay CV of 12% and 10%; IL-6 (ml058097)—the standard curve of 10 to 400 pg/mL and inter- and intra-assay CV of 12% and 10%; TNF-α (ml002360)–the standard curve of 10 to 200 pg/mL and inter- and intra-assay CV of 12% and 10%, respectively.

### 2.8. Serum Antioxidative Levels

The activities of glutathione peroxidase (GSH-Px, A006-2-1), total antioxidant capacity (T-AOC, A015-2-1), catalase (CAT, A007-1-1), and superoxide dismutase (SOD, A001-3-2) and the level of malondialdehyde (MAD, A003-1-2) were measured by commercial antioxidant kits (Nanjing Jiancheng Bioengineering Institute, Nanjing, China).

### 2.9. Intestinal Histomorphologic Analysis

Intestinal histopathology analyses were performed according to the hematoxylin–eosin (HE) staining method [17]. After fixation for 24 h, the duodenum, jejunum, and ileum samples were paraffin-embedded, made into 5 µm sections, and stained with hematoxylin and eosin. Stained intestinal tissue sections were measured by villus height and crypt depth under computer-assisted microscopy (Leica DMI3000B microscopy, Wetzlar, Germany). Ten villi and corresponding crypts were selected from each section per piglet, and the villus-height-to-crypt-depth ratio was measured.

### 2.10. RNA Extraction and Quantitative Real-Time PCR

Trizol (MAN0001271, Invitrogen, Carlsbad, CA, USA) was used to extract total RNA in the frozen intestinal tissues (duodenum, jejunum, ileum, and colon). The concentration and reversed transcriptive process of total RNA were assessed and performed using the nucleic acid concentration detector (Eppendorf AG, Hamburg, Germany) and cDNA Synthesis Kit (Code No. 6210A, Takara, Otsu, Japan) according to the manufacturers’ instructions. Real-time quantitative PCR was performed as previously described by Bai et al. (2020) [18]. A LightCycler^®^ 480 Real-Time PCR instrument (Roche, Basel, Switzerland) was used for PCR amplification. The primes of relevant gene sequences used for qPCR amplification were designed by Primer Premier 6.0, as shown in Table S1. The relative levels of target genes were calculated using the Ct value (2^−ΔΔCt^) method after normalization with β-actin.

### 2.11. Colonic Bacterial 16S rDNA Gene Sequencing and Function Prediction

The total DNA of colonic contents was extracted using the PowerFecal DNA Isolation kit (Megan, Guangzhou, China). During the sample cracking process, all samples (*n* = 6 per group) needed to be repeatedly knocked for complete bacterial DNA extraction. The quantity and quality of all extracted DNA samples were determined using 0.80% agarose gel electrophoresis and Qubit dsDNA kit (Q32851, Thermo Fisher Scientific, Waltham, MA, USA) for 16S sequencing. 16S sequencing and library preparation were conducted by Novogene Bioinformatics Technology Co., Ltd. [19]. Based on the 16S rRNA gene data, functional profiles of the microbial community were predicated using the PICRUSt software package. Based on 16S RNA datasets and mapping files, the high-level phenotypes of colonic microbiota were measured by Bugbase [20]. Sequences of the 16S rRNA gene were submitted to the NCBI Sequence Read Archive database under accession number PRJNA1131608.

### 2.12. Detection of Short-Chain Fatty Acids (SCFAs) in Colonic Contents

Colonic contents were collected and freeze-dried to determine short-chain fatty acids (SCFAs). The contents of acetic, propionic, butyric, isobutyric, valeric, and isopentanoic acids were analyzed using Agilent 6890 gas chromatography (Agilent Technologies, Inc., Palo Alto, CA, USA). About 0.5 g of frozen dried colonic contents were homogenized with water in a 15 mL centrifuge tube, centrifuged at 10,000× *g* and 4 °C for 10 min, and transferred the supernatant into a 10 mL centrifuge tube. Mixed with 25% metaphosphoric acid solution at 9:1, the supernatant was placed at room temperature for 4 h and filtered through a 0.45 µM polysulfone filter for analysis.

### 2.13. Statistical Analysis

Experimental results were analyzed using IBM SPSS Statistics 26 software (SPSS Inc., Chicago, IL, USA) and represented by mean ± standard error of the mean (SEM). The difference among four treatments was compared using one-way ANOVA and Duncan’s Multiple-Range Test (*n* = 8). Based on regressions of estimation curves, we the determined linear and quadratic models for the dietary AR supplementation dose. GraphPad Prism 8.0 (GraphPad Software, Inc., La Jolla, CA, USA) was used to make figures. Kruskal–Wallis analysis was conducted on 16S rRNA sequencing data to compare treatment group differences using nonparametric tests. The results of statistical significance are shown as *p* <  0.05, and 0.05 < *p*  <  0.10 showed a significant trend.

## 3. Results

### 3.1. Growth Performance

The growth performance of weaned piglets is shown in Table 3. Piglets fed with the MAR diet had a higher (*p* < 0.05) ADG than those fed with the BD and HAR diets. In both the MAR and HAR groups, the ADFI of piglets significantly increased (*p* < 0.05) compared to the BD and LAR groups. There was no significant effect on the final body weight or F/G ratio among the four groups (*p* > 0.05). Moreover, dietary AR supplementation had a significant linear effect on ADFI (*p* = 0.019, linear model: Y = 637.17 + 30.87 X, R^2^ = 0.470).

### 3.2. Serum Biochemical Indices

To evaluate the changes in body metabolic status following AR treatments, we tested the serum biochemical indices in the weaned piglets (Table 4). LAR and MAR showed significantly higher serum TP concentrations than the BD group (*p* < 0.05), while no marked change was observed in the HAR group (*p* > 0.05). And the MAR group had a higher serum TP concentration than the LAR group (*p* < 0.05). The serum C3 level was markedly increased in all AR-treated groups (*p* < 0.05). The regression analysis showed that serum C3 levels increased significantly (*p* = 0.033) and that 3.0% AR was the optimal level for supplementation (Y = 0.023 + 0.012 X − 0.002 X2, R^2^ = 0.209).

### 3.3. Serum Immune Indexes

Levels of immunoglobulins and cytokines in the serum of weaned piglets are shown in Table 5. Supplementation with AR significantly increased (*p* < 0.05) the serum IgA level compared to the BD group. The MAR and HAR diets significantly decreased (*p* < 0.05) the level of IL-6 in serum compared to the BD group. Regression analysis showed that the serum IgA level increased quadratically (Y = 38.688 + 9.636 X − 1.946 X2, *p* = 0.012, R^2^ = 0.422), indicating that the highest level was associated with 2.47% AR. There was a significant linear and quadratic relationship between dietary AR supplementation and serum IL-6 level (*p* = 0.010, linear model: Y = 848.762 − 60.472 X, R^2^ = 0.464; *p* = 0.018, quadratic model: Y = 882.617 − 154.093 X + 22.895 X2; estimated optimal dose was 3.36% AR, R^2^ = 0.236).

### 3.4. Serum Antioxidant Indices

The effect of AR on the antioxidant ability of weaned piglets is presented in Table 6. The diet supplemented with AR had a higher serum SOD activity in weaned piglets than the BD group (*p* < 0.05). There was a significant linear and quadratic relationship between dietary AR supplementation and serum SOD activity (*p* = 0.003, linear model: Y = 9.379 + 0.862 X, R^2^ = 0.270; *p* < 0.001, quadratic model: Y = 8.287 + 3.205 X − 0.574 X2; estimated optimal dose required was 2.79% AR, R^2^ = 0.451).

### 3.5. Intestinal Histomorphologic Analysis

**Figure 1:**
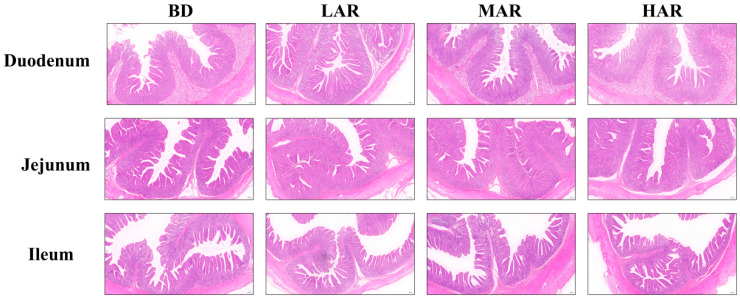
The intestinal morphology was histologically analyzed by hematoxylin and eosin (HE, 200 μm). BD, control group, basal diet without antibiotics; LAR, the control diet + 1% *Artemisia annua* residue; MAR, the control diet + 2% *Artemisia annua* residue; HAR, the control diet + 4% *Artemisia annua* residue. Data are expressed as means ± SEM (*n* = 8).

The supplementation of AR affected intestinal morphology as shown in Table 7 and Figure 1. The AR treatments significantly increased the villus height in the duodenum and crypt depth in the jejunum (*p* < 0.05). Compared with the BD and LAR groups, the MAR and HAR groups increased the villus height in the jejunum (*p* < 0.05) and decreased the crypt depth in the ileum (*p* < 0.05). The LAR and HAR groups significantly increased the crypt depth and the ratio of villus height/crypt depth in the duodenum compared to the BD group (*p* < 0.05). The MAR group had the highest ratio of villus height/crypt depth in the ileum among the four treatments (*p* < 0.05). Compared with the BD and MAR groups, the LAR group decreased the ratio of villus height/crypt depth in the jejunum (*p* < 0.05).

Regression analysis showed that there were significant linear and quadratic effects of dietary AR treatment on the duodenal villus height (*p* < 0.001, linear model: Y = 429.709 + 20.507 X, R^2^ = 0.118; *p* < 0.001, quadratic model: Y = 410.959 + 63.644 X − 10.673 X2, with an estimated optimal dose of 2.98%, R^2^ = 0.162), crypt depth (*p* = 0.023, linear model: Y = 254.463 + 8.023 X, R^2^ = 0.197; *p* = 0.046, quadratic model: Y = 249.506 + 19.428 X − 2.822 X2, with an estimated optimal dose of 3.44%, R^2^ = 0.161), and the ratio of villus height/crypt depth (*p* = 0.034, linear model: Y = 1.15 + 2.16 X, R^2^ = 0.324; *p* = 0.048, quadratic model: Y = 1.740 + 0.171 X − 0.028 X2, with an estimated optimal dose of 3.05%, R^2^ = 0.411), jejunal villus height (*p* < 0.001, linear model: Y = 372.818 + 22.424 X, R^2^ = 0.128; *p* < 0.001, quadratic model: Y = 376.153 + 15.154 X − 1.784 X2, with an estimated optimal dose of 4.24%, R^2^ = 0.129), crypt depth (*p* < 0.001, linear model: Y = 196.330 + 13.490 X, R^2^ = 0.195; *p* < 0.001, quadratic model: Y = 194.313 + 17.886 X − 1.079 X2, with an estimated optimal dose of 8.29%, R^2^ = 0.196), and ileal crypt depth (*p* < 0.001, linear model: Y = 260.924 − 9.509 X, R^2^ = 0.155; *p* < 0.001, quadratic model: Y = 271.023 − 31.519 X + 5.314 X2, with an estimated optimal dose of 2.96%, R^2^ = 0.151). A quadratic model was fitted to the villus height/crypt depth ratio (*p* = 0.0009, Y = 1.700 + 0.269 X − 0.059 X2, R^2^ = 0.167), indicating that 2.80% AR supplementation was optimal.

### 3.6. Expression of Genes Associated with Intestinal Mucosal Barrier

**Figure 2:**
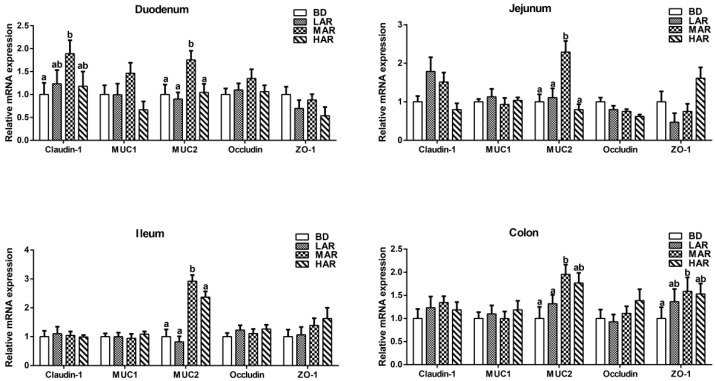
Effects of dietary *Artemisia annua* residue on gene expression levels of the intestinal mucosal barrier of weaned piglets. BD, control group, basal diet without antibiotics; LAR, the control diet + 1% *Artemisia annua* residue; MAR, the control diet + 2% *Artemisia annua* residue; HAR, the control diet + 4% *Artemisia annua* residue. Data are expressed as means ± SEM (*n* = 8). Means with different superscripts in the columns are significantly different (*p* < 0.05).

The gene expression levels associated with the intestinal mucosal barrier of weaned piglets are shown in Figure 2. The MAR diet had the highest (*p* < 0.05) mRNA expression level of MUC2 in the mucosa of duodenum, jejunum, and ileum among all groups. Compared to the BD group, the MAR remarkably increased (*p* < 0.05) the mRNA expression level of Claudin-1 in the duodenum and ZO-1 in the colon. And MAR increased (*p* < 0.05) the MUC2 mRNA expression level in the colon compared to the BD and LAR groups.

As shown in Table 8, in a regression analysis, there were significant linear and quadratic effects of dietary AR on jejunal occludin mRNA expression (*p* = 0.019, linear model: Y = 0.942 − 0.077 X, R^2^ = 0.231; *p* = 0.049, quadratic model: Y = 0.983 − 0.163 X + 0.021 X2, with an estimated optimal dose of 3.88% AR, R^2^ = 0.221), ileal MUC2 mRNA expression (*p* = 0.005, linear model: Y = 0.721 + 0.637 X, R^2^ = 0.200; *p* = 0.019, quadratic model: Y = 0.872 − 0.320 X + 0.077 X2, with an estimated optimal dose of 2.08%, R^2^ = 0.222), and colonic MUC2 mRNA expression (*p* = 0.016, linear model: Y = 0.732 + 0.426 X, R^2^ = 0.276; *p* = 0.026, quadratic model: Y = 0.776 − 0.350 X + 0.046 X2, with an estimated optimal dose of 3.80%, R^2^ = 0.282).

### 3.7. Colonic Microbiological Compositions and Metabolic Functions

**Figure 3:**
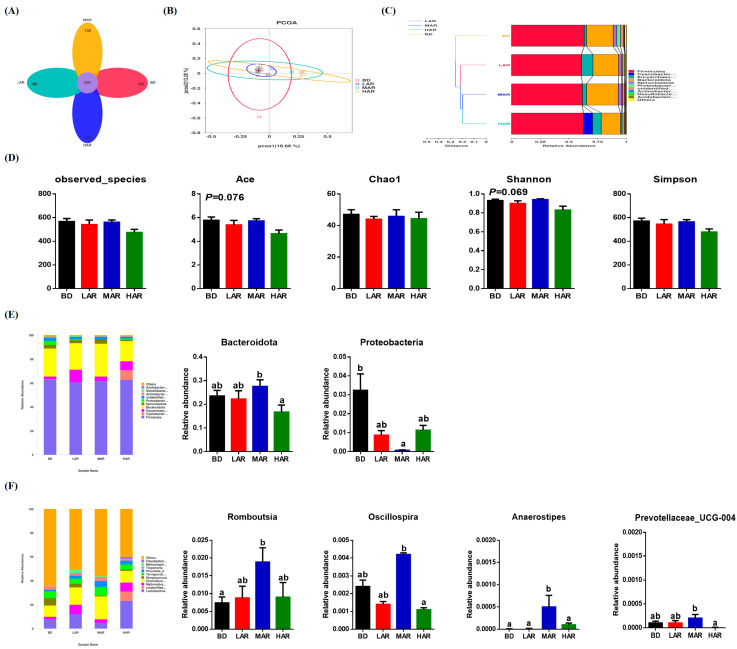
Effect of dietary *Artemisia annua* residue on the colonic microbiota diversity and composition in the weaning pigs. (**A**) A Venn diagram illustrating the overlaps of OTUs in the gut microbiota; (**B**) principal coordinate analysis (PCoA); (**C**) unweighted unifrac cluster tree based on Unweighted Pair-group Method with Arithmetic Mean (UPGMA) analysis; (**D**) the microbial alpha diversity indexes (Observed-species, Ace, Shannon, Simpson, Chao1) were calculated using the mothur program; (**E**) relative contribution of the top 10 phyla in each group (left) and the relative abundance of significantly different microorganisms (right); (**F**) relative contribution of the top 10 genera in each group (left) and the relative abundance of significantly different microorganisms (right). BD, control group, basal diet without antibiotics; LAR, the control diet + 1% *Artemisia annua* residue; MAR, the control diet + 2% *Artemisia annua* residue; HAR, the control diet + 4% *Artemisia annua* residue. Data are expressed as means ± SEM (*n* = 6). Means with different superscripts in the columns are significantly different (*p* < 0.05).

To confirm the effects of AR on intestinal flora, a Venn diagram was created and the differences in richness among treatments are shown in Figure 3A. Four treatments contained 658 common OTUs, while BD, LAR, MAR, and HAR contained 443, 189, 133, and 162 unique OTUs, respectively. Based on the unweighted pair-group method with arithmetic mean (UPGMA) analysis, the principal coordinate analysis (PCoA, Figure 3B) of β-diversity and the unweighted Unifrac cluster tree (Figure 3C) showed the difference, similarity, and phylogeny in the microbiota among the four treatments. The intestinal microbial α-diversity is shown in Figure 3D, and the microbial richness indices (ACE) and the diversity indices (Shannon) had a significant increasing trend (0.05 < *p* < 0.10). As shown in Figure 3E, at the phylum level, MAR significantly increased the relative abundance of *Bacteroidota* in the colon compared to the HAR group (*p* < 0.05). Compared with the BD group, MAR decreased the relative abundance of *Proteobacteria* in the colon (*p* < 0.05). At the genus level, MAR significantly increased the relative abundance of *Romboutsia* in the colon compared to the BD group (*p* < 0.05). BD had the highest relative abundance of *Anaerostipes* among all groups (*p* < 0.05). MAR had higher relative abundances of *Oscillospira* and *Prevotellaceae_UCG-004* in the colon than the HAR group (*p* < 0.05).

**Figure 4:**
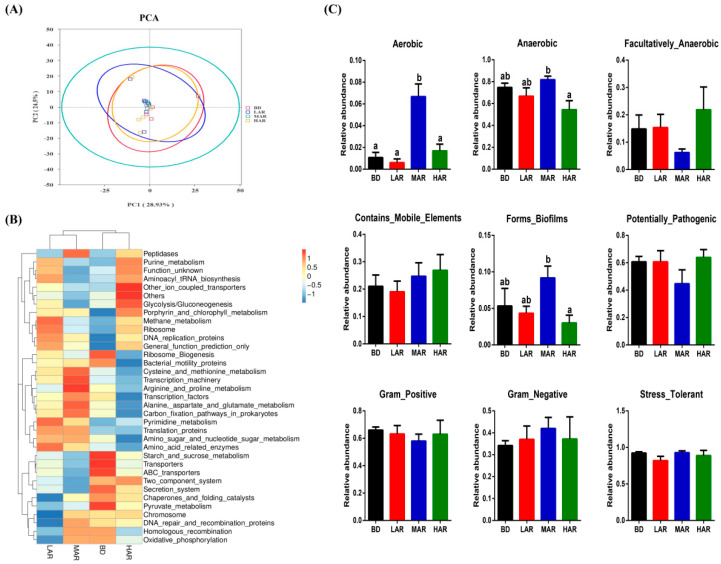
*Artemisia annua* residue altered the metabolic functions and phenotypes of colonic microbiota in the weaning pigs. (**A**) Principal components analysis (PCA) of functional profiles in the gut microbiota; (**B**) heatmap tree based on different metabolism-related pathways at KEGG level 3; (**C**) The metabolic phenotype predictions were compared using BugBase online (https://bugbase.cs.umn.edu/). BD, control group, basal diet without antibiotics; LAR, the control diet + 1% *Artemisia annua* residue; MAR, the control diet + 2% *Artemisia annua* residue; HAR, the control diet + 4% *Artemisia annua* residue. Data are expressed as means ± SEM (*n* = 6). Means with different superscripts in the columns are significantly different (*p* < 0.05).

The effects of dietary AR supplementation on the metabolic functions of gut microbiota are shown in Figure 4. The PCA revealed a significant separation among the four treatments (Figure 4A). Based on different microbial-metabolism-related pathways at the KEGG level 3, the heap map showed that LAR significantly increased Pyrimidine_metabolism, Amino_acid_related_enzymes, DNA_replication_proteins, and Ribosome_Biogenesis pathways; MAR increased Cysteine_and_methionine_metabolism, Transcription_machinery, Arginine_and_proline_metabolism, Arginine_and_proline_metabolism, and Carbon_fixation_pathways_in_prokaryotes pathways; and HAR increased the Glycolysis/Gluconeogenesis and Other_ion_coupled_transporters pathways compared to other three groups (Figure 4B). In terms of Bugbase analysis, the MAR diet had the highest aerobic bacterial richness among all treatments (*p* < 0.05). Compared with the HAR group, the richness of anaerobic bacteria and biofilm-forming showed a marked increase by MAR treatment (*p* < 0.05).

### 3.8. Colonic SCFA Contents

Table 9 reveals the differences in colonic SCFA concentrations among all treatments. The MAR group had the highest concentration of acetic acid in the colon among the four treatments (*p* < 0.05). Compared with the BD group, MAR significantly increased the concentration of butyric acid (*p* < 0.05). No significant difference was observed in the concentrations of propionic acid, valeric acid, isobutyric acid, and isopentanoic acid of colonic contents (*p* > 0.05) among all groups. There were significant quadratic relationships between dietary AR supplementation and colonic acetic acid content (*p* = 0.013, quadratic model: Y = 1892.561 + 439.560 X − 100.589 X2, with an estimated optimal dose of 2.18%, R^2^ = 0.218) and butyric acid content (*p* = 0.046, quadratic model: Y = 651.533 + 202.399 X − 46.004 X2, with an estimated optimal dose of 2.20%, R^2^ = 0.116).

## 4. Discussion

Pig producers generally consider weaning to be one of the most crucial periods in pig production, which directly impacts the industry’s economic performance [21]. Enhancing immunity and antioxidant capacity and strengthening intestinal barrier function are effective ways to reduce the occurrence of weaning stress and improve animal production, thereby highlighting the need for nutritional interventions in further studies [22,23]. Experiments demonstrated that *Artemisia annua* has anti-inflammatory and anti-oxidant effects in animal production [24,25]. The addition of *Artemisia annua* L. to the diet could improve the growth performance of piglets [4,26], geese [3], and broilers [27]. This is similar to our study, where the 2% AR-supplemented diet significantly increased the ADG and ADFI in weaned piglets. The possible reason for the improvement in growth performance of weaned piglets is that most of the active components and nutrients retained by AR, which can play an antibacterial role, regulate the immune system and promote intestinal health, so as to improve the utilization rate of the diet. In addition, the current results proved the beneficial influences of AR supplementation in improving weaned piglets’ growth performance, immunity, antioxidant capacity, and intestinal barrier function.

As an imperative parameter to reflect the physiological and pathological changes of the body, serum biochemical indices are usually determined to evaluate the growth performance of animals. Serum TP mainly contains albumin and globulin, which directly reflect the protein metabolism level and immune function in the body [28]. In this study, dietary supplementation with 2% AR increased the serum TP, indicating that AR treatment could strengthen the body’s protein synthesis capacity of weaned piglets. Serum C3, as an enzyme-active globulin, plays a key role in immune regulation, clearing immune complexes, stabilizing the internal environment of the body, and participating in allergic reactions and autoimmune diseases [29]. Serum IgA is a crucial immunoglobulin in humoral immunity, which is involved in the first line of defense against pathogen invasion [30]. Therefore, the higher the contents of serum C3 and IgA are, the stronger the body’s immunity. The changes in inflammatory cytokine levels such as TNF-α, IL-1β, and IL-6 are important indicators for assessing inflammation. In this study, dietary AR addition increased the serum C3 and IgA contents while decreasing the Il-6 level. This result shows that AR supplementation contributes to improving the body’s immune index and reducing disease occurrence in weaned piglets. Song et al. (2018) also reported that enzymatically treated *Artemisia annua* increased the serum IgA and reduced the pro-inflammatory cytokines of heat-stressed broilers [31]. Similarly, Cui et al. (2024) showed that *Artemisia annua* increased the immunoglobulins’ IgA level and reduced the IL-6 level in the serum of geese [3]. In addition, Xiong et al. (2022) found that enzymatically treated *Artemisia annua* could inhibit inflammatory cytokines levels by regulating MAPK and NF-κB in heat-stressed sows [26]. Ethanol extract of *Artemisia annua* could inhibit the NF-κB pathway to down-regulate CD36 and IL-6 expressions in bovine mammary epithelial cells [32]. It was concluded that AR can enhance the immunity of weaned piglets, and the specific mechanism needs to be verified in future studies.

Oxidative stress is increased in piglets during the weaning period, which is caused by an imbalance between reactive oxygen species and antioxidant systems [33]. Enhanced antioxidative enzyme activities can improve the body’s defense function against oxidative damage. There was a decrease in the activities of T-AOC, SOD, and GSH-Px and an increase in the content of MDA in the serum of weaned piglets [34,35]. Antioxidant enzymes can remove excess oxygen free radicals to reduce the body’s oxidative damage. *Artemisia annua* has abundant antioxidant bioactive constituents such as polyphenols and flavonoids that might have antioxidant functions for livestock and poultry [36]. Dietary *Artemisia annua* supplementation increased serum T-AOC, SOD, and GSH-Px activities and decreased MDA content in broilers [37,38]. Similar results were observed in the present study; weaned piglets fed with AR had higher serum SOD activity, which might be due to the presence of flavonoids and polyphenols in AR. Collectively, AR had the potential to be a protein source feed to prevent oxidative stress by activating the enzymatic antioxidant defense system in piglets during the weaning process.

The development of intestinal villi in weaned piglets plays a crucial role in reducing intestinal inflammation and improving growth. Increasing the villus height contributes to providing a greater absorption surface area, and the lower crypt depth reflects the stronger rapid self-renewal and repair ability of intestinal stem cells [39]. Previous studies found that 1% *Artemisia annua* increased the villus height/crypt depth ratio and decreased the crypt depth in the jejunum of geese [3]. In our study, 2% AR supplementation increased the duodenal and jejunal villus height and the ileal villus height/crypt depth ratio and decreased the ileal crypt depth. The results indicate that AR could promote the jejunal and ileal morphology development in weaned piglets, which may be an important reason for improving growth performance. Moreover, the integrity of the intestinal barrier is directly related to intestinal inflammation and diarrhea in weaned piglets [40]. Weaning stress often damages intestinal structural integrity and barrier function in piglets [41]. Tight junctions contain cytosolic proteins, zonula occludens (ZO-1), and transmembrane protein complexes (occludins and claudins), as crucial structures to maintain intestinal barrier function and health [42,43]. Because piglets’ intestinal development is incomplete, intestinal tight junctions’ protein expression decreased under weaning stress [44]. The current study found that dietary AR improved the duodenal Claudin-1 and colonic ZO-1 and MUC2 expression levels in weaned piglets. Previous studies also found that enzymatically treated *Artemisia annua* L. increased intestinal occludin and claudin-1 levels in piglets [24]. During nutrient starvation in jejunal IPEC-J2 cells, tight junctions may undergo remodeling and endocytosis, resulting in impaired intercellular barrier function [45]. It is speculated that AR diets may enhance the intestinal barrier function by relieving nutrient starvation and enhancing intestinal tight junctions. The MUC2 barrier can directly prevent intestinal bacteria, which play a crucial role in intestinal health [46]. In addition, studies have found that IL-6 and IFN-γ as pro-inflammatory cytokines increased intestinal permeability by decreasing the levels of ZO-1 and MUC2 [47]. Collectively, AR could promote intestinal barrier function by improving tight junction protein levels in weaned piglets.

The intestinal microbiota can regulate the metabolism and health and affect the intestinal barrier function. The structure and composition of intestinal microbiota are related to the digestion/absorption capacity, the body’s antioxidants, and the body’s immune system [48,49]. The increase in microbial diversity can increase the resistance to intestinal pathogen colonization and reduce the appearance of intestinal diseases [50], and the present research indicated that dietary AR had a significant tendency to increase microbial diversity in weaned piglets. Various studies have reported that *Artemisia annua* L. could regulate intestinal microbiota [51,52] and increase microbial diversity [3]. In terms of species composition, 2% AR addition increased the abundance of phyla Bacteroidota and decreased the abundance of phyla Proteobacteria, and the phyla Bacteroidota as the main species contributes to nutrient absorption and metabolism [53]. This is consistent with Cui et al. (2024), who found that *Artemisia annua* increased the relative abundance of Bacteroidota in the cecum of geese [3]. Increased Bacteroidota also contributes to maintaining microbiota homeostasis, regulating the immune system and promoting nutrient absorption in the intestine [54]. Higher Bacteroidota is beneficial to improving the immune system and preventing diarrhea in weaned piglets [55]. *Proteobacteria* is commonly used as a microbial signature of disease, mainly involving inflammatory bowel disease and metabolic disorders, which is a positive association with IL-6 and IFN-γ [56]. At the genus level, 2% AR supplementation showed higher abundances of *Romboutsia*, *Oscillospira*, and *Prevotellaceae_UCG-004*, and *Romboutsia* and *Oscillospira* as important genera of intestinal bacteria can promote the production of butyrate and inhibit inflammation [57,58]. *Prevotellaceae UCG-004* has been proven to be positively correlated with carbohydrate metabolism and the production of SCFA [59]. This further explains how dietary AR increased SCFA content in the colon, especially acetic acid and butyric acid. However, existing studies also found that the addition of *Artemisia annua* had beneficial effects on intestinal health by increasing Fecalibacterium and Paraprevotella richness in the cecum of geese [3]. The inconsistencies between these results may be related to the differences in the experimental animal, intestinal segments, and active components.

Meanwhile, combined with microbial function prediction, the present results further indicate that dietary AR may promote amino acid metabolism and DNA synthesis in gut microbial communities to improve weaned piglets’ growth performance. We first evaluated the influences of AR diets on microbial phenotypes in weaned piglets. The present study showed that AR supplementation increased the richness of biofilm-forming and anaerobic bacteria. Microbial biofilm formation and anaerobic bacteria richness have been negatively correlated with inflammation and the development of drug resistance and pathogenesis [60]. Intestinal SCFAs are involved in the energy supply of intestinal epithelial cells, affecting intestinal cavity PH and electrolyte balance, mucosal barrier permeability and sensitivity, etc., which are closely related to intestinal diseases [61]. In the current study, 2% AR supplementation increased colonic acetic acid and butyric acid concentrations. Consistent with the previous study, the *Artemisia annua* diet could enrich the diversity of intestinal flora and increase the production of SCFAs, especially acetic acid and butyric acid [5]. The increased production of acetic acid by intestinal microorganisms had a significant protective effect on intestinal inflammation [62]. As a mucus layer and immunological development enhancer, butyric acid inhibits intestinal pathogenic microorganisms [63]. Therefore, based on previous research, we speculated that AR may improve growth performance and immunity by regulating the intestinal microbial structure and metabolisms in weaned piglets, and the correlation analysis among indicators needs to be further explored.

Developing AR as an unconventional feed resource in pig production has considerable theoretical and economic value and significant social benefits. In the current research, we paid more attention to the beneficial effects of AR on antioxidant, immune, and microbial regulation in weaned piglets while lacking the detection of digestion and utilization. Thus, it is crucial to explore the mechanism of AR to enhance immunity and improve intestinal health by combining the digestive and metabolic characteristics and the composition of effective active ingredients in future studies.

## 5. Conclusions

In summary, this study provided compelling evidence that AR could improve growth performance, antioxidant ability, and immunity and enhance the intestinal barrier function of piglets during the weaning period. Adding an appropriate amount of dietary AR increased the serum IgA content and SOD activity and intestinal tight junction protein (ZO-1 and Claudin-1) and MUC2 expressions and improved intestinal morphology, microbial structure, and SCFA production to improve the growth of weaned piglets. In conclusion, AR has potential as an unconventional feed material in piglet production, and 2.08 to 4.24% dietary AR for weaned piglets is theoretically recommended.

## Figures and Tables

**Table 1:** Nutritional value and main active components of *Artemisia annua* residue.

Items	Calculation Based on Sample Weight	Calculation Based on Dry Matter
Nutritional values ^1^		
Moisture (%)	6.19	
Dry matter (%)	93.81	
Crude ash (%)	13.15	14.01
Fat (%)	1.51	1.61
Crude protein (%)	18.44	19.65
Acid detergent lignin (%)	4.50	4.80
Crude fiber (%)	13.02	13.88
Acid detergent fiber (%)	26.50	28.25
Neutral detergent fiber (%)	55.04	58.66
Main active components ^2^		
	Retention Time, Molecular weight	Relative content (air-dry basis, %)
Alcohol extract of *Artemisia annua* L. residue		
Rosmarinic acid	22.54, 359.077	13.71
Chrysosplenetin B	23.957, 373.0928	3.61
Scopoletin	14.115, 191.0349	3.52
Homovanillic acid	18.766, 181.0506	2.35
Water extract of *Artemisia annua* L. residue		
Quinic acid	1.453, 150.053	4.86
Cryptochlorogenic acid	10.495, 354.095	3.55
3-O-Caffeoylquinic acid methyl ester	12.242, 368.109	5.16
Artemisinin	18.777, 282.146	12.33

^1^ The nutritional values were measured. Moisture (method GB/T 6435-2014), dry matter (method GB/T 6435-2014), ash (method GB/T 6438-2007), fat (method GB/T 6433-2006), crude protein (method GB/T 6432-2018), acid detergent lignin (method GB/T 20805-2006), crude fiber (method GB/T 6434-2022), neutral detergent fiber (method GB/T 20806-2022), and acid detergent fiber (method NY/T 1459-2022) were determined. ^2^ The main active components of AR were determined by High-Performance Liquid Chromatography (HPLC).

**Table 2:** Composition and calculated nutrient levels of the basal diet (air-dry basis).

Ingredients	Content (%)	Calculated Nutrient Levels	Content
Corn (4.52% crude protein)	48.97	Digestible energy (MJ/kg)	14.75
Soybean meal (43% crude protein)	13.40	Crude protein (%)	17.10
Puffing corn powder	10.00	Calcium (%)	0.43
Extruded soybean	10.00	Total phosphorus (%)	0.63
Soybean oil	4.00	Available phosphorus (%)	0.36
Fish meal	6.00	Lysine (%)	1.22
Whey powder	4.00	Methionine (%)	0.38
Monocalcium phosphate	0.60	Methionine + cysteine (%)	0.66
Antioxidants	0.20	Analyzed nutrient levels ^2^	
Limestone	0.70	Gross energy (MJ/kg)	16.32
Salt	0.60	Crude protein	17.60
Lysine (98%)	0.30	Calcium	0.48
Methionine	0.15	Total phosphorous	0.66
Threonine	0.08	Lysine	1.28
Premix ^1^	1.00	Methionine	0.32
		Methionine + cysteine	0.64
Total	100.00		

^1^ Provided per kilogram of diet: vitamin A, 8000 IU; vitamin D3, 2000 IU; vitamin E, 300 mg; vitamin K, 30 mg; vitamin B1, 30 mg; vitamin B2, 60 mg; vitamin B6, 30 mg; biotin, 0.2 mg; folic acid, 10 mg; niacin, 300 mg; pantothenic acid, 300 mg; Cu (CuSO_4_.5H_2_O), 190 mg; Fe (FeSO_4_.H_2_O), 190 mg; Mn (MnSO_4_.H_2_O), 45 mg; Se (NaSeO_3_), 0.40 mg; Zn (ZnO), 140 mg; I (Ca(IO_3_)_2_), 0.5 mg. ^2^ Gross energy was measured by an Isothermal Automatic Heat Meter (5E-AC8018, Kaide Automatic Equipment Changsha Co., Ltd., Changsha, China). Dry matter (method GB/T 6435-2014), crude protein (method GB/T 6432-2018), calcium (method GB/T 13885-2017), phosphorus (method GB/T 6437-2002), and amino acids (Lysine, Methionine and Cysteine, method GB/T 18246-2019) were determined.

**Table 3:** Effects of dietary *Artemisia annua* residue on the growth performance of weaned piglets.

Items ^2^	Groups ^1^				*p* Values		
	BD	LAR	MAR	HAR	AR	Linear	Quadratic
Initial body weight, kg	7.48 ± 0.27	7.56 ± 0.59	7.76 ± 0.46	7.32 ± 0.64	0.988	0.835	0.914
Final body weight, kg	12.36 ± 0.43	13.40 ± 0.80	15.04 ± 1.05	12.08 ± 1.03	0.188	0.557	0.501
ADG, g/d	212.17 ± 27.22 ^a^	253.91 ± 58.14 ^ab^	316.52 ± 54.17 ^b^	206.96 ± 64.90 ^ac^	0.017	0.647	0.827
ADFI, g/d	588.36 ± 101.93 ^a^	648.54 ± 91.91 ^a^	730.30 ± 90.55 ^b^	719.42 ± 96.88 ^b^	0.014	0.019	0.064
F/G ratio	2.80 ± 0.36	2.70 ± 0.64	2.30 ± 0.43	3.90 ± 1.90	0.123	0.190	0.427

^1^ BD, control group, basal diet without antibiotics; LAR, the control diet + 1% *Artemisia annua* residue; MAR, the control diet + 2% *Artemisia annua* residue; HAR, the control diet + 4% *Artemisia annua* residue. ^2^ ADG, average daily gain; ADFI, average daily feed intake; F/G ratio, feed/gain ratio. Data are expressed as means ± SEM (*n* = 8). Means within a row with different superscripts are significantly different (*p* < 0.05).

**Table 4:** Effects of dietary *Artemisia annua* residue on the serum biochemical indices of weaned piglets.

Items ^2^	Groups ^1^				*p* Values		
	BD	LAR	MAR	HAR	AR	Linear	Quadratic
TP, g/L	54.29 ± 1.10 ^a^	60.88 ± 2.52 ^bc^	61.88 ± 1.55 ^c^	55.68 ± 2.40 ^ab^	0.026	0.509	0.149
ALB, g/L	31.30 ± 1.90	35.56 ± 1.53	35.36 ± 1.73	34.39 ± 2.69	0.426	0.431	0.293
GLU, mmol/L	5.43 ± 0.21	6.11 ± 0.18	6.04 ± 0.27	5.74 ± 0.28	0.183	0.648	0.123
TG, mmol/L	0.64 ± 0.07	0.71 ± 0.06	0.80 ± 0.08	0.83 ± 0.11	0.324	0.080	0.179
CHOL, mmol/L	2.96 ± 0.13	3.29 ± 0.19	3.31 ± 0.18	3.30 ± 0.20	0.431	0.241	0.275
LDL-C, mmol/L	1.72 ± 0.09	1.97 ± 0.12	1.83 ± 0.17	1.87 ± 0.15	0.631	0.676	0.737
HDL-C, mmol/L	1.34 ± 0.05	1.44 ± 0.11	1.51 ± 0.13	1.53 ± 0.09	0.502	0.160	0.302
ALT, U/L	82.93 ± 6.99	93.84 ± 8.62	111.3 ± 12.59	93.93 ± 8.16	0.216	0.413	0.136
AST, U/L	43.63 ± 4.04	52 ± 4.88	53.75 ± 5.13	56.25 ± 4.40	0.264	0.077	0.143
ALP, U/L	274.75 ± 29.48	274.75 ± 19.69	306.38 ± 28.46	300.75 ± 29.59	0.766	0.402	0.662
UN, nmol/mL	4.24 ± 0.45	4.53 ± 0.64	4.4 ± 0.49	3.45 ± 0.45	0.461	0.203	0.270
C3, g/L	0.02 ± 0 ^a^	0.03 ± 0 ^b^	0.04 ± 0 ^b^	0.03 ± 0 ^b^	0.011	0.097	0.033
C4, g/L	0.03 ± 0	0.04 ± 0	0.03 ± 0	0.03 ± 0	0.600	0.969	0.602

^1^ BD, control group, basal diet without antibiotics; LAR, the control diet + 1% *Artemisia annua* residue; MAR, the control diet + 2% *Artemisia annua* residue; HAR, the control diet + 4% *Artemisia annua* residue. ^2^ TP, total protein; TG, triglyceride; GLU, glucose; CHOL, cholesterol; HDL-C, high-density lipoprotein; LDL-C, low-density lipoprotein; UN, urea nitrogen; C3, complement component 3; C4, complement component 4; AST, aspartate aminotransferase; ALP, alkaline phosphatase; ALT, alanine aminotransferase. Data are expressed as means ± SEM (*n* = 8). Means within a row with different superscripts are significantly different (*p* < 0.05).

**Table 5:** Effects of dietary *Artemisia annua* residue on serum immune indices of weaned piglets.

Items ^2^	Groups ^1^				*p* Values		
	BD	LAR	MAR	HAR	AR	Linear	Quadratic
IgA, µg/mL	38.29 ± 1.47 ^a^	45.58 ± 1.26 ^b^	49.56 ± 1.34 ^b^	44.59 ± 2.69 ^b^	0.001	0.094	0.012
IgG, µg/mL	438.3 ± 27.48	438.55 ± 20.9	430.67 ± 15.88	442.43 ± 25.67	0.987	0.072	0.158
IgM, µg/mL	51.63 ± 2.47	51.93 ± 2.86	54.91 ± 2.23	52.66 ± 1.61	0.752	0.821	0.928
IL-1β, ng/mL	55.94 ± 3.22	51.9 ± 1.99	54.37 ± 3.03	54.4 ± 1.85	0.745	0.769	0.953
IL-6, ng/mL	868.25 ± 85.92 ^b^	797.4 ± 62.95 ^ab^	622.91 ± 19.67 ^a^	638.31 ± 20.55 ^a^	0.032	0.010	0.018
TNF-α, pg/mL	480.24 ± 31.28	449.94 ± 22.7	441.34 ± 13.06	453.5 ± 31.28	0.736	0.531	0.526

^1^ BD, control group, basal diet without antibiotics; LAR, the control diet + 1% *Artemisia annua* residue; MAR, the control diet + 2% *Artemisia annua* residue; HAR, the control diet + 4% *Artemisia annua* residue. ^2^ IgA, immunoglobulin A; IgG, immunoglobulin G; IgM, immunoglobulin M; IL-1β, interleukin 1β; IL-6, interleukin 6; TNF-α, tumor necrosis factor-α. Data are expressed as means ± SEM (*n* = 8). Means within a row with different superscripts are significantly different (*p* < 0.05).

**Table 6:** Effects of dietary Artemisia annua residue on serum antioxidant indices of weaned piglets.

Items ^2^	Groups ^1^				*p* Values		
	BD	LAR	MAR	HAR	AR	Linear	Quadratic
T-AOC, U/mL	0.54 ± 0.02	0.56 ± 0.04	0.47 ± 0.04	0.51 ± 0.04	0.295	0.382	0.499
GSH-Px, U/mL	241.68 ± 22.87	246.13 ± 27.93	228.43 ± 23.84	218.47 ± 8.4	0.820	0.374	0.676
CAT, U/mL	0.01 ± 0.002	0.01 ± 0.002	0.01 ± 0.002	0.004 ± 0	0.333	0.114	0.126
SOD, U/mL	8.07 ± 0.77 ^a^	11.67 ± 0.38 ^b^	11.96 ± 0.49 ^b^	11.96 ± 0.83 ^b^	<0.001	0.003	<0.001
MAD, nmol/mL	24.59 ± 0.47	24.25 ± 0.36	23.77 ± 0.21	24.02 ± 0.29	0.396	0.343	0.258

^1^ BD, control group, basal diet without antibiotics; LAR, the control diet + 1% *Artemisia annua* residue; MAR, the control diet + 2% *Artemisia annua* residue; HAR, the control diet + 4% *Artemisia annua* residue. ^2^ T-AOC, total antioxidant capacity; GSH-Px, glutathione peroxidase; CAT, catalase; SOD, superoxide dismutase; MAD, malondialdehyde. Data are expressed as means ± SEM (*n* = 8). Means within a row with different superscripts are significantly different (*p* < 0.05).

**Table 7:** Effects of dietary *Artemisia annua* residue on the intestinal morphology of weaned piglets.

Items	Groups ^1^				*p* Values		
	BD	LAR	MAR	HAR	AR	Linear	Quadratic
Duodenum							
Villus height, μm	389.89 ± 12.80 ^a^	542.6 ± 17.05 ^d^	449.67 ± 12.73 ^b^	503.37 ± 13.76 ^c^	<0.001	<0.001	<0.001
Crypt depth, μm	241.42 ± 8.45 ^a^	296.3 ± 12.97 ^c^	259.46 ± 8.53 ^ab^	285.37 ± 12 ^bc^	0.001	0.023	0.046
Villus height/crypt depth	1.7 ± 0.05^a^	2.03 ± 0.10 ^b^	1.88 ± 0.07 ^ab^	1.99 ± 0.10 ^b^	0.016	0.034	0.048
Jejunum							
Villus height, μm	382.23 ± 9.92 ^a^	370.53 ± 8.47 ^a^	427.7 ± 10.01 ^b^	462.66 ± 11.52 ^c^	<0.001	<0.001	<0.001
Crypt depth, μm	189.56 ± 6.53 ^a^	227.3 ± 6.84 ^b^	215.66 ± 6.34 ^b^	250.5 ± 8.77 ^c^	<0.001	<0.001	<0.001
Villus height/crypt depth	2.26 ± 0.11 ^b^	1.7 ± 0.08 ^a^	2.06 ± 0.08 ^b^	2 ± 0.09 ^ab^	0.001	0.267	0.136
Ileum							
Villus height, μm	445.47 ± 6.74	428.99 ± 5.85	407.14 ± 9.17	392.36 ± 10.39	0.265	0.126	0.214
Crypt depth, μm	265.39 ± 5.79 ^b^	252.84 ± 5.99 ^b^	214.42 ± 7.72 ^a^	230.77 ± 6.51 ^a^	<0.001	<0.001	<0.001
Villus height/crypt depth	1.76 ± 0.05 ^a^	1.79 ± 0.05 ^a^	2.07 ± 0.12 ^b^	1.84 ± 0.05 ^a^	0.032	0.323	0.009

^1^ BD, control group, basal diet without antibiotics; LAR, the control diet + 1% *Artemisia annua* residue; MAR, the control diet + 2% *Artemisia annua* residue; HAR, the control diet + 4% *Artemisia annua* residue. Data are expressed as means ± SEM (*n* = 8). Means within a row with different superscripts are significantly different (*p* < 0.05).

**Table 8:** Gene expression levels of the intestinal mucosal barrier.

Items (Y)	Correlation, *p*-Value	Optimum Addition Amount of *Artemisia annua* Residue (X, %)
Jejunum		
Occludin mRNA expression	Linear model: Y = 0.942 − 0.077 X, *p* = 0.019 Quadratic model: Y = 0.021 X2 − 0.163 X + 0.983, *p* = 0.049	3.88
Ileum		
MUC2 mRNA expression	Linear model: Y = 0.721 + 0.637 X, *p* = 0.005 Quadratic model: Y = 0.077 X2 − 0.320 X + 0.872, *p* = 0.019	2.08
Colon		
MUC2 mRNA expression	Linear model: Y = 0.732 + 0.426 X, *p* = 0.016 Quadratic model: Y = 0.046 X2 − 0.350 X + 0.776, *p* = 0.026	3.80

**Table 9:** Effects of dietary *Artemisia annua* residue on colonic short-chain fatty acid contents of weaned piglets.

Items	Groups ^1^				*p* Values		
	BD	LAR	MAR	HAR	AR	Linear	Quadratic
Acetic acid, µg/g	1954.47 ± 101.530 ^a^	2042.85 ± 92.29 ^a^	2493.15 ± 140.96 ^b^	2017.79 ± 138.51 ^a^	0.005	0.524	0.013
Propionic acid, µg/g	935.81 ± 64.82	964.80 ± 61.46	1031.80 ± 64.52	992.85 ± 103.38	0.810	0.532	0.656
Butyric acid, µg/g	677.71 ± 53.52 ^a^	728.14 ± 57.33 ^ab^	924.67 ± 57.95 ^b^	715.08 ± 94.63 ^ab^	0.039	0.549	0.046
Valeric acid, µg/g	129.58 ± 15.01	182.81 ± 29.67	161.95 ± 23.60	130.31 ± 16.66	0.251	0.831	0.497
Isobutyric acid, µg/g	55.22 ± 3.06	58.84 ± 5.99	54.32 ± 4.98	55.23 ± 7.14	0.938	0.864	0.981
Isopentanoic acid, µg/g	126.31 ± 11.32	147.28 ± 14.69	149.33 ± 10.51	126.19 ± 17.58	0.466	0.658	0.719

^1^ BD, control group, basal diet without antibiotics; LAR, the control diet + 1% *Artemisia annua* residue; MAR, the control diet + 2% *Artemisia annua* residue; HAR, the control diet + 4% *Artemisia annua* residue. Data are expressed as means ± SEM (*n* = 8). Means within a row with different superscripts are significantly different (*p* < 0.05).

## Data Availability

The original contributions presented in the study are included in the article; further inquiries can be directed to the corresponding authors.

## Supplementary Materials

The following supporting information can be downloaded at: https://www.mdpi.com/article/10.3390/ani14243569/s1, Table S1: Primers used for quantitative real-time polymerase chain reaction.
