# Supplementary material for: Artemisia annua Residue Regulates Immunity, Antioxidant Ability, Intestinal Barrier Function, and Microbial Structure in Weaned Piglets
Source: Animals (Basel). 2024 Dec 10;14(24):3569. doi: 10.3390/ani14243569 (PMC11672813; doi:10.3390/ani14243569)
Supplement: Supplementary file 1 [file animals-14-03569-s001.zip › animals-3345680-supplementary.pdf]

Table S1 Primers used for quantitative real-time polymerase chain reaction

Gene	Accession No.	Primer, 5′–3′	Size (bp)	T_A (°C)
β-actin	XM_021086047.1	F: CTGCGGCATCCACGAAACT	147	61
		R: AGGGCCGTGATCTCCTTCTG		
Claudin-1	NM_001244539.1	F: AAGGACAAAACCGTGTGGGA	247	60
		R: CTCTCCCCACATTCGAGATGATT		
Occludin	NM_001163647.2	F:ACGAGCTGGAGGAAGACTGGATC	238	60
		R:CCCTTAACTTGCTTCAGTCTATTG		
ZO-1	XM_021098896.1	F: CCTGCTTCTCCAAAAACTCTT	252	60
		R: TTCTATGGAGCTCAACACCC		
MUC1	XM_021089729.1	F: AGATCCCACCACCAGCTACT	95	59
		R: AAAAGAGTCCCAGAAGCCCG		
MUC2	XM_021082584.1	F: GGACGACACCATCTACCTCAC	149	60
		R: TGTTCCACACGAGAGCAAGG		

T_A, annealing temperature; ZO-1 = zonula occludens-1; MUC1, mucin 1; MUC2, mucin 2.
